# Supplementary material for: Effect of Diurnal Variation of Heart Rate and Respiratory Rate on Activation of Rapid Response System and Clinical Outcome in Hospitalized Children
Source: Children (Basel). 2023 Jan 14;10(1):167. doi: 10.3390/children10010167 (PMC9857164; doi:10.3390/children10010167)
Supplement: Supplementary file 1 [file children-10-00167-s001.zip › children-2044211-supplementary.pdf]

Table S1. Distribution of underlying diseases among study subjects

Disease category	No. of patients (%)
Behavioral disorder	180 (0.7)
Congenital and genetic disorder	4,670 (17.3)
Dermatologic disease	398 (1.5)
Endocrinologic disease	1,679 (6.2)
Gastrointestinal disease	2,591 (9.6)
Genitourinary disease	1,254 (4.7)
Hemato-oncologic disease	5,665 (21)
Infectious disease	703 (2.6)
Injury and intoxication	899 (3.3)
Musculoskeletal disease	1,347 (5)
Neurologic disease	2,126 (7.9)
Ophthalmic and otologic disease	1,031 (3.8)
Others	4,380 (16.3)

All diseases diagnosed by the patient are included, and there may be more than one diagnosis pertaining to an individual.

Table S2. Heart rate centiles by age during the daytime (from 8:00 to 20:00)

Age	Centile												
	1st	3rd	5th	10th	15th	25th	50th	75th	85th	90th	95th	97th	99th
0–<3 months	118	123	127	131	135	140	149	158	162	166	171	174	180
3–<6 months	112	117	120	124	127	132	140	149	153	156	161	164	169
6–<9 months	104	110	113	117	120	125	133	141	146	149	153	156	162
9–<12 months	101	106	109	114	117	121	129	138	142	145	150	153	158
12–<15 months	99	104	107	112	115	119	128	136	140	143	148	151	156
15–<18 months	98	103	106	110	113	118	126	135	139	142	146	149	155
18–<21 months	96	102	105	109	112	116	125	133	137	140	145	148	153
21–<24 months	94	100	103	107	110	115	123	131	136	139	143	146	152
2–<3 years	93	98	101	105	108	113	121	129	134	137	141	144	150
3–<4 years	86	91	94	98	101	106	114	122	127	130	134	137	143
4–<5 years	81	86	89	94	97	101	109	117	122	125	129	132	138
5–<6 years	78	83	86	91	94	98	106	114	119	122	126	129	135
6–<7 years	76	81	84	89	92	96	104	112	117	120	124	127	133
7–<8 years	74	80	83	87	90	94	103	111	115	118	123	125	131
8–<9 years	73	78	81	85	88	93	101	109	114	117	121	124	129
9–<10 years	71	77	79	84	87	91	99	108	112	115	119	122	128
10–<11 years	69	75	78	82	85	90	98	106	110	113	118	121	126
11–<12 years	68	73	76	80	83	88	96	104	109	112	116	119	124
12–<13 years	66	71	74	79	82	86	94	102	107	110	114	117	122
13–<14 years	64	70	73	77	80	84	92	101	105	108	112	115	121
14–<15 years	62	68	71	75	78	82	91	99	103	106	111	113	119
15–<16 years	61	66	69	73	76	81	89	97	101	104	109	112	117
16–<17 years	59	64	67	72	74	79	87	95	100	103	107	110	115
17–<18 years	57	62	65	70	73	77	85	93	98	101	105	108	113

Table S3. Heart rate centiles by age during the nighttime (from 20:00 to 8:00 the next day)

Age	Centile												
	1st	3rd	5th	10th	15th	25th	50th	75th	85th	90th	95th	97th	99th
0–<3 months	112	118	122	127	131	137	147	157	162	166	171	175	181
3–<6 months	103	109	112	117	120	125	134	143	148	151	156	159	165
6–<9 months	96	102	105	110	113	118	127	135	140	143	148	151	157
9–<12 months	91	97	100	105	108	113	122	131	135	139	143	146	152
12–<15 months	88	94	97	102	105	110	119	128	132	136	140	144	149
15–<18 months	87	93	96	100	104	108	117	126	131	134	139	142	148
18–<21 months	85	91	94	99	102	107	116	125	129	133	137	140	146
21–<24 months	84	90	93	98	101	106	114	123	128	131	136	139	145
2–<3 years	83	89	92	96	100	104	113	122	127	130	135	138	143
3–<4 years	77	83	86	91	94	99	107	116	121	124	129	132	137
4–<5 years	72	78	81	86	89	94	102	111	116	119	124	127	133
5–<6 years	69	75	78	83	86	91	99	108	113	116	121	124	129
6–<7 years	68	74	77	81	84	89	98	106	111	114	119	122	128
7–<8 years	67	72	75	80	83	88	97	105	110	113	118	121	126
8–<9 years	66	71	74	79	82	87	96	104	109	112	117	120	125
9–<10 years	65	70	73	78	81	86	94	103	108	111	115	118	124
10–<11 years	63	69	72	77	80	84	93	102	106	109	114	117	123
11–<12 years	62	67	70	75	78	83	91	100	105	108	113	116	121
12–<13 years	60	66	69	73	77	81	90	98	103	106	111	114	120
13–<14 years	58	64	67	72	75	79	88	97	101	104	109	112	118
14–<15 years	57	62	65	70	73	78	86	95	99	103	107	110	116
15–<16 years	55	61	64	68	71	76	84	93	98	101	105	108	114
16–<17 years	53	59	62	66	70	74	83	91	96	99	104	107	112
17–<18 years	51	57	60	65	68	72	81	90	94	97	102	105	111

Table S4. Respiratory rate centiles by age during the daytime (from 8:00 to 20:00)

Age	Centile												
	1st	3rd	5th	10th	15th	25th	50th	75th	85th	90th	95th	97th	99th
0–<3 months	26	28	30	32	33	35	39	43	45	47	49	50	53
3–<6 months	23	25	27	29	30	32	36	39	41	42	44	46	48
6–<9 months	22	24	25	27	28	30	33	37	39	40	42	43	45
9–<12 months	21	23	24	26	27	29	32	35	37	38	40	41	43
12–<15 months	20	22	23	25	26	28	31	34	35	36	38	39	41
15–<18 months	20	22	23	24	25	27	30	33	34	35	37	38	40
18–<21 months	20	21	22	24	25	26	29	32	33	34	36	37	38
21–<24 months	19	21	22	23	24	26	28	31	32	33	35	36	37
2–<3 years	19	20	21	22	23	24	26	29	30	31	32	33	34
3–<4 years	18	20	20	21	22	23	25	27	28	29	30	31	32
4–<5 years	18	19	20	21	22	22	24	26	27	28	29	29	31
5–<6 years	18	19	20	20	21	22	24	25	26	27	28	28	29
6–<7 years	18	19	19	20	21	21	23	25	25	26	27	27	28
7–<8 years	18	19	19	20	20	21	23	24	25	25	26	27	27
8–<9 years	17	18	19	20	20	21	22	23	24	25	25	26	27
9–<10 years	17	18	19	19	20	20	22	23	24	24	25	25	26
10–<11 years	17	18	18	19	20	20	21	23	23	24	24	25	26
11–<12 years	17	18	18	19	19	20	21	22	23	23	24	24	25
12–<13 years	17	18	18	19	19	20	21	22	23	23	24	24	25
13–<14 years	17	18	18	19	19	20	21	22	22	23	23	24	24
14–<15 years	17	18	18	19	19	19	20	22	22	22	23	23	24
15–<16 years	17	18	18	18	19	19	20	21	22	22	23	23	24
16–<17 years	17	18	18	18	19	19	20	21	22	22	22	23	23
17–<18 years	17	17	18	18	19	19	20	21	21	22	22	23	23

Table S5. Respiratory rate centiles by age during the nighttime (from 20:00 to 8:00 the next day)

Age	Centile												
	1st	3rd	5th	10th	15th	25th	50th	75th	85th	90th	95th	97th	99th
0–<3 months	32	35	37	39	40	43	47	51	54	55	57	59	62
3–<6 months	25	27	29	31	32	35	39	43	45	47	49	50	53
6–<9 months	22	25	26	28	29	31	35	39	41	42	44	45	48
9–<12 months	21	23	25	26	28	29	33	36	38	39	41	42	44
12–<15 months	21	23	24	25	26	28	31	34	36	37	39	40	42
15–<18 months	20	22	23	25	26	27	30	33	35	36	37	38	40
18–<21 months	20	22	22	24	25	26	29	32	33	34	36	37	39
21–<24 months	19	21	22	23	24	26	28	31	32	33	35	36	38
2–<3 years	19	21	22	23	24	25	28	30	32	33	34	35	36
3–<4 years	19	20	21	22	23	24	26	28	29	30	31	32	34
4–<5 years	18	19	20	21	22	23	25	27	28	29	30	30	32
5–<6 years	18	19	20	21	21	22	24	26	27	27	28	29	30
6–<7 years	18	19	19	20	21	22	23	25	26	26	27	28	29
7–<8 years	18	19	19	20	20	21	23	24	25	26	27	27	28
8–<9 years	17	18	19	20	20	21	22	24	25	25	26	26	27
9–<10 years	17	18	19	19	20	21	22	23	24	25	25	26	27
10–<11 years	17	18	18	19	20	20	22	23	24	24	25	25	26
11–<12 years	17	18	18	19	19	20	21	23	23	24	24	25	26
12–<13 years	17	18	18	19	19	20	21	22	23	23	24	24	25
13–<14 years	17	18	18	19	19	20	21	22	23	23	24	24	25
14–<15 years	17	18	18	19	19	20	21	22	22	23	23	24	24
15–<16 years	17	17	18	18	19	19	20	22	22	22	23	23	24
16–<17 years	17	17	18	18	19	19	20	21	22	22	23	23	24
17–<18 years	17	17	18	18	19	19	20	21	22	22	23	23	24

Table S6. Regression analyses between underlying diseases and z-scores differences of HR and RR by age

Variables	Z-score of HR by age			Z-score of RR by age		
	Estimate	SE	*P*	Estimate	SE	*P*
Others	Reference			Reference		
Behavioral disorder	0.073	0.052	0.159	0.069	0.039	0.074
Congenital & genetic disorder	0.089	0.023	<0.001	0.033	0.017	0.059
Dermatologic disease	-0.059	0.035	0.094	0.026	0.026	0.319
Endocrinologic disease	0.004	0.02	0.828	0.030	0.015	0.048
Gastrointestinal disease	0.040	0.018	0.026	0.024	0.013	0.072
Hemato-oncologic disease	0.062	0.012	<0.001	0.012	0.009	0.174
Infectious disease	0.069	0.028	0.012	0.009	0.021	0.668
Injury & intoxication	-0.006	0.025	0.793	0.017	0.018	0.346
Musculoskeletal disease	-0.004	0.021	0.852	0.022	0.016	0.168
Neurologic disease	-0.006	0.018	0.732	0.016	0.013	0.233
Ophthalmic & otologic disease	-0.074	0.025	0.003	0.008	0.019	0.687

The difference of each measurement was defined as the nighttime measurement minus the daytime measurement. Daytime was defined as 8:00 to 20:00, and nighttime was defined as 20:00 to 8:00 the next day.

HR = heart rate, RR = respiratory rate, SE = standard error

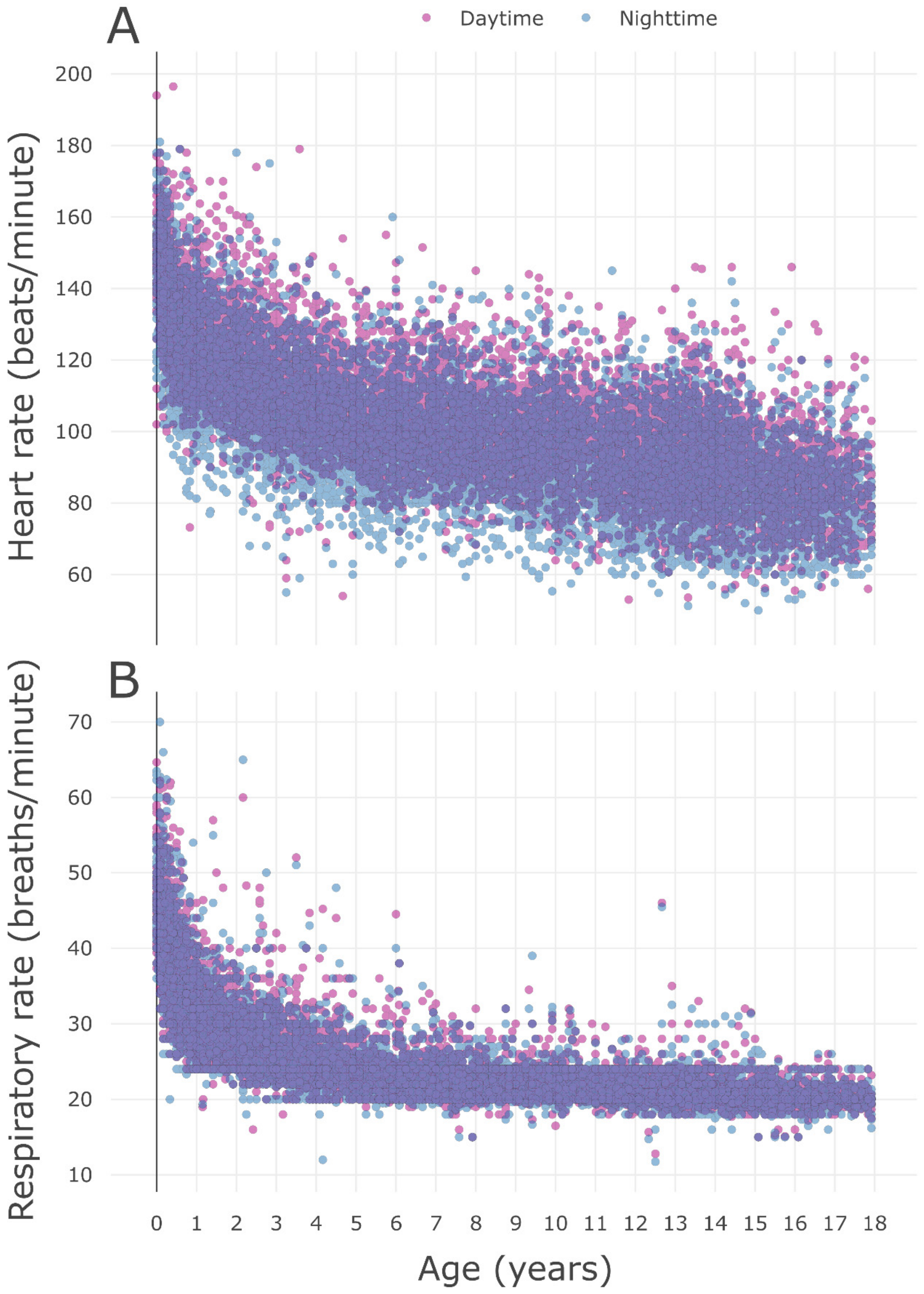

Figure S1. Scatter plot of vital signs by time period
The distribution of (A) heart rate and (B) respiratory rate is shown. Daytime was defined as 8:00 to 20:00, and nighttime was defined as 20:00 to 8:00 the next day.

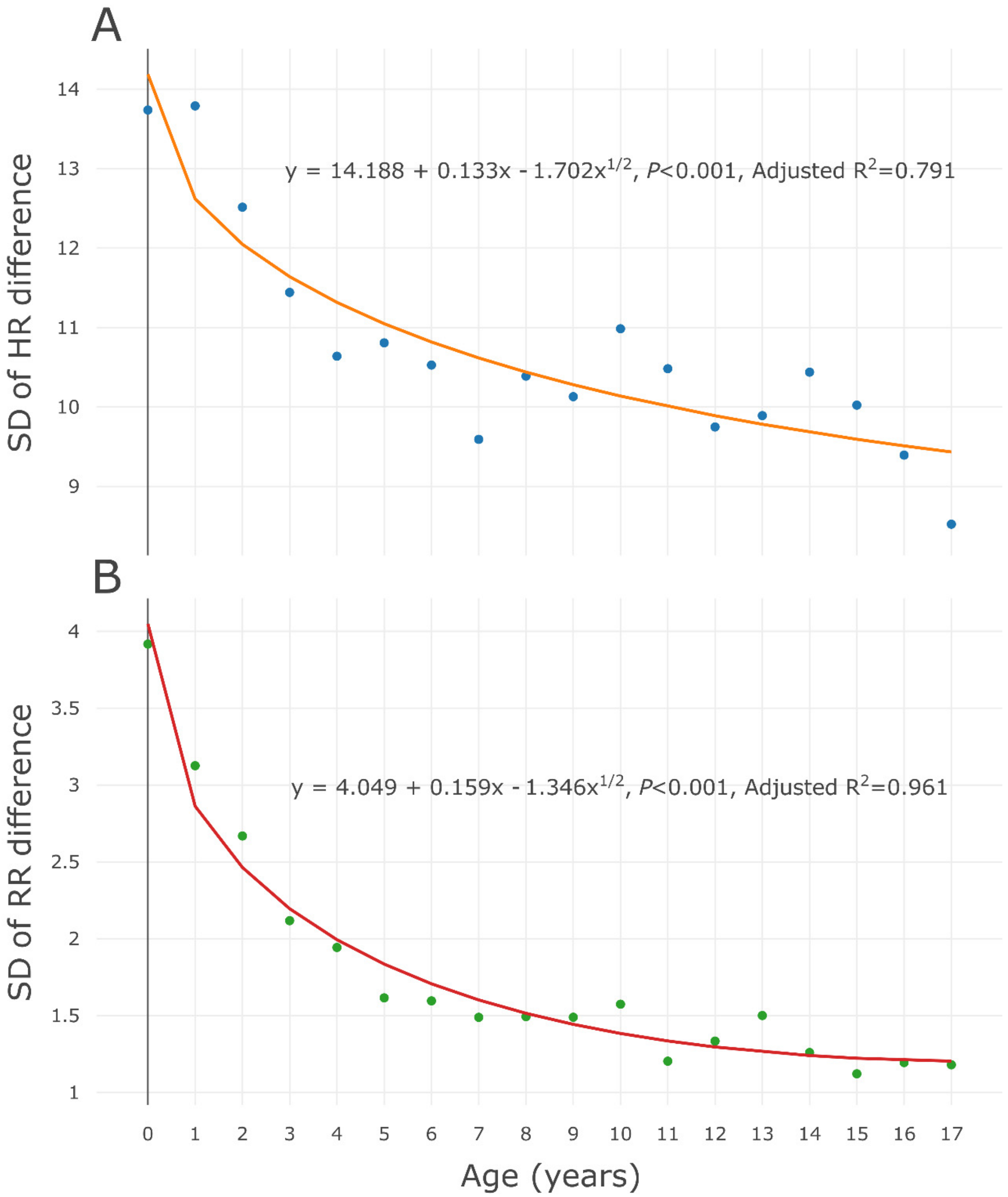

Figure S2. SD changes of differences in vital signs by age

The difference in vital signs was defined as the value obtained by subtracting the measured value of daytime from the measured value of nighttime. Nighttime was defined as from 8:00 pm to 8:00 am the next day, and daytime was defined as from 8:00 am to 8:00 pm. (A) SD of HR difference by age, and (B) SD of RR difference. Both HR and RR differences show a statistically significant decrease as age increases.

SD = standard deviation, HR = heart rate, RR = respiratory rate

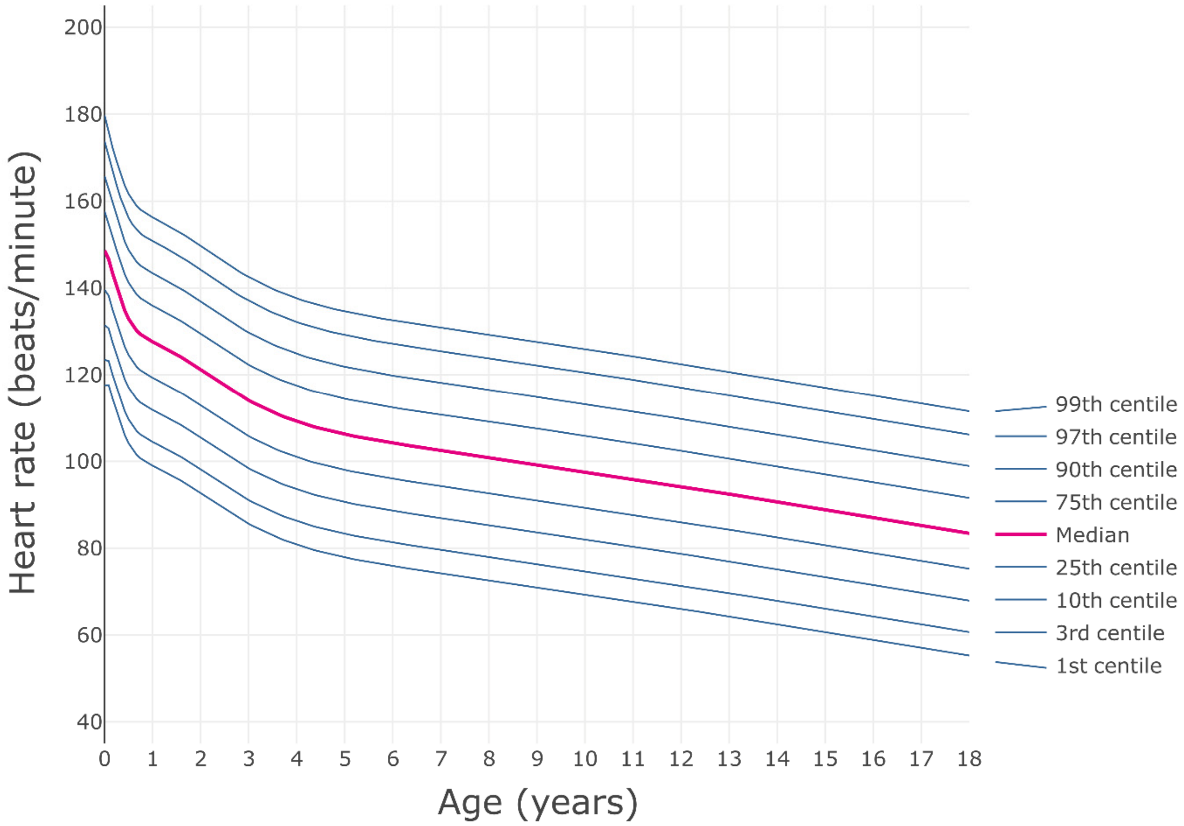

Figure S3. Centile curve of heart rate by age in the daytime (from 8:00 to 20:00)

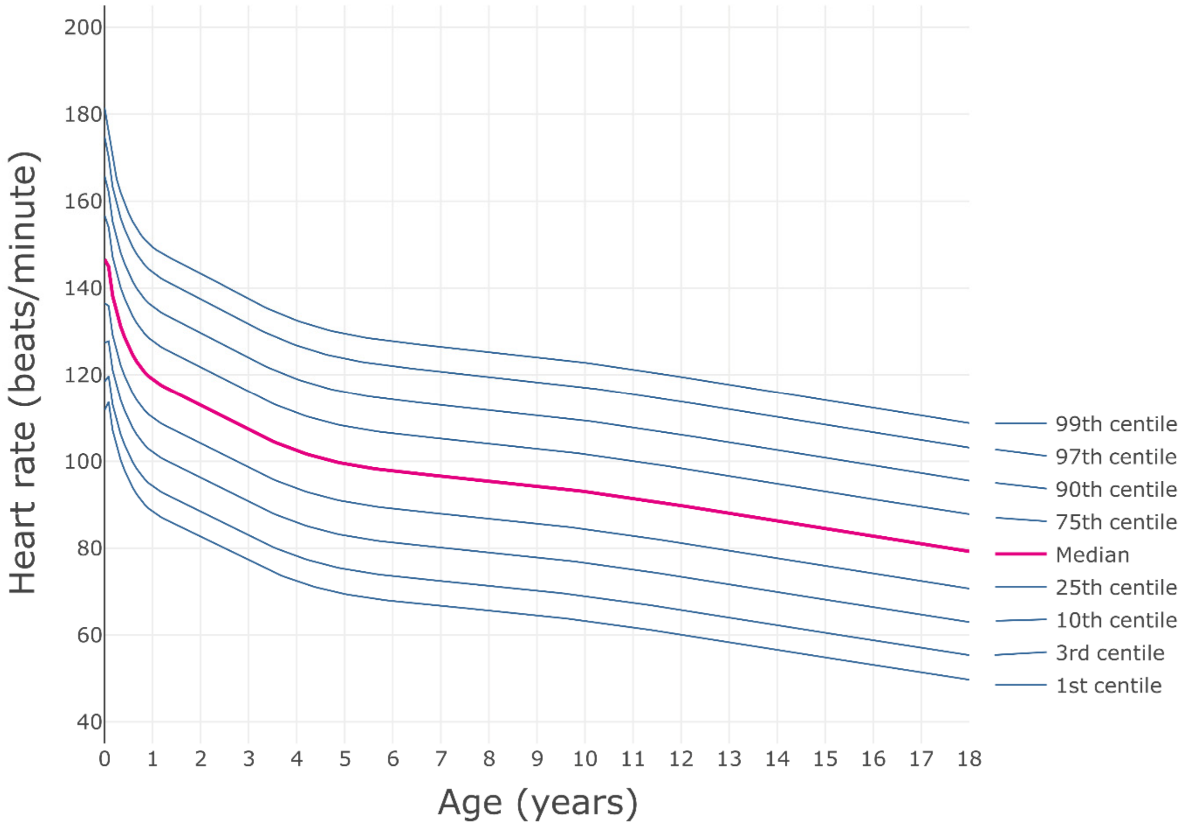

Figure S4. Centile curve of heart rate by age in the nighttime (from 20:00 to 8:00 the next day)

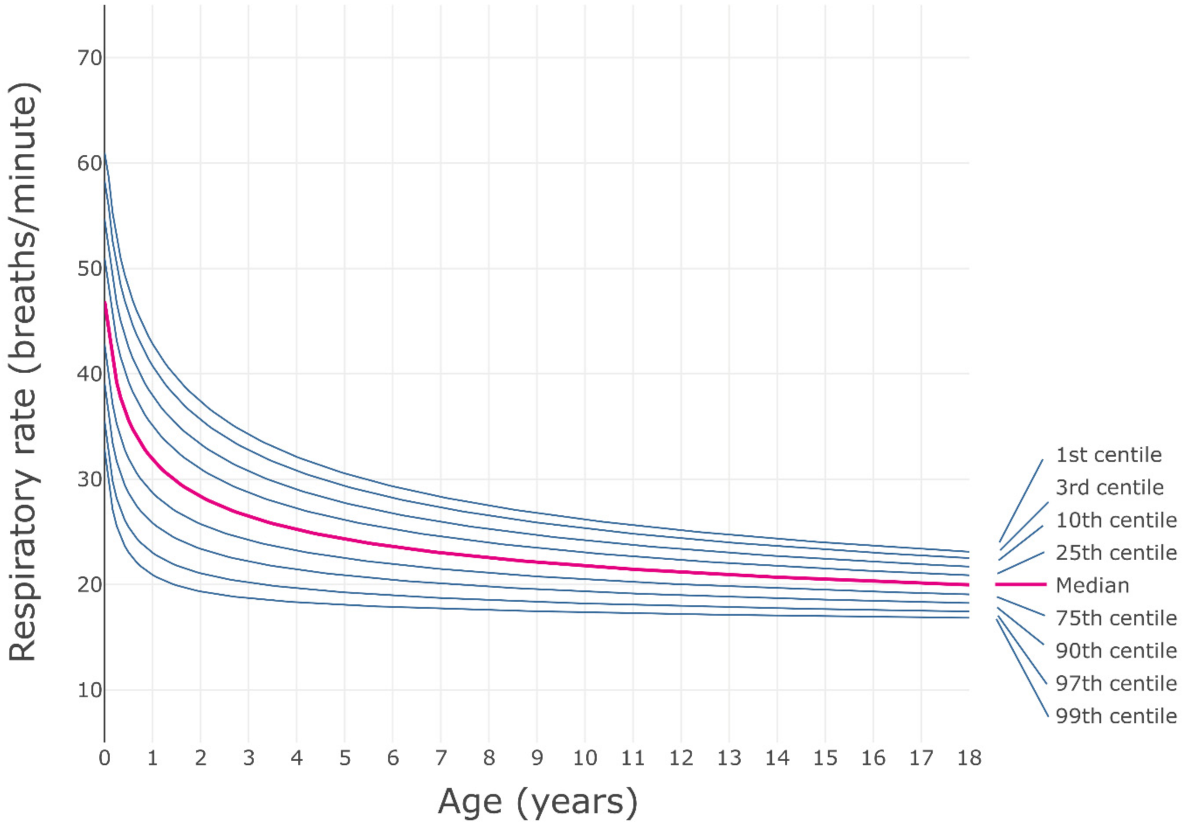

Figure S5. Centile curve of respiratory rate by age in the daytime (from 8:00 to 20:00)

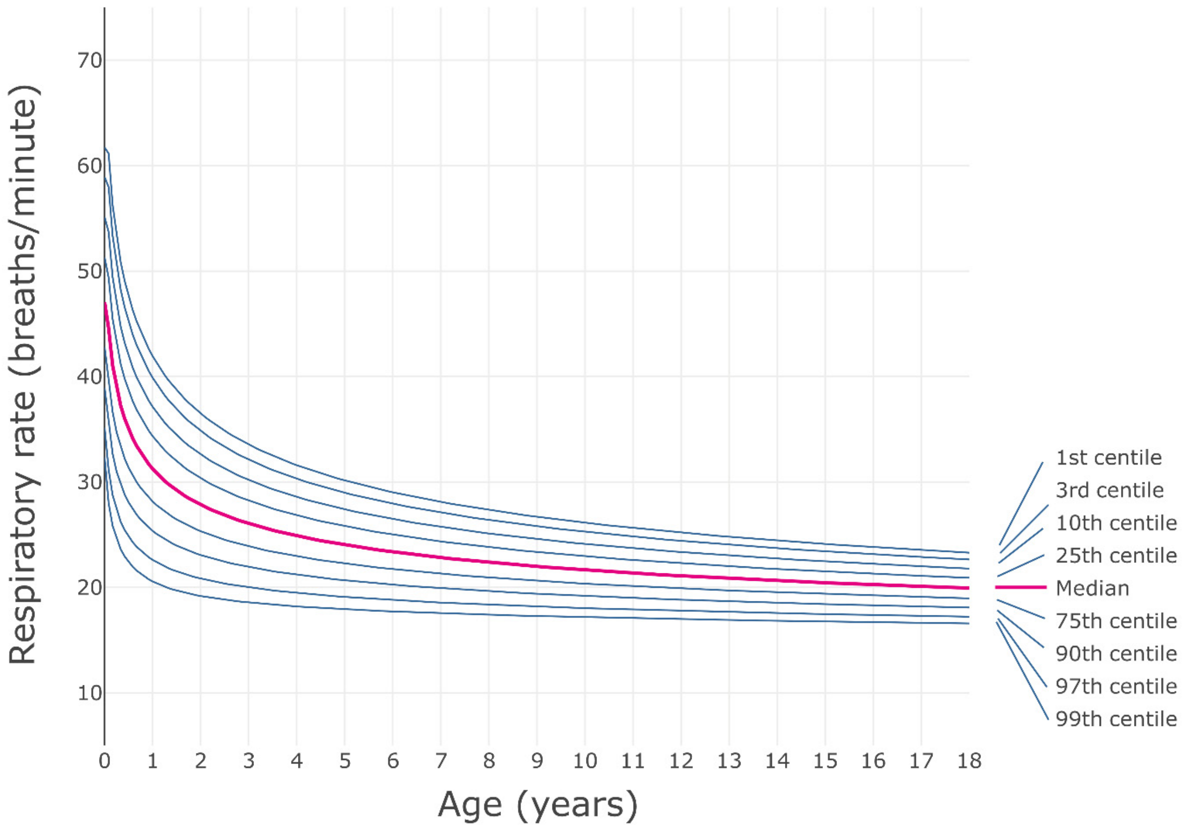

Figure S6. Centile curve of respiratory rate by age in the nighttime (from 20:00 to 8:00 the next day)

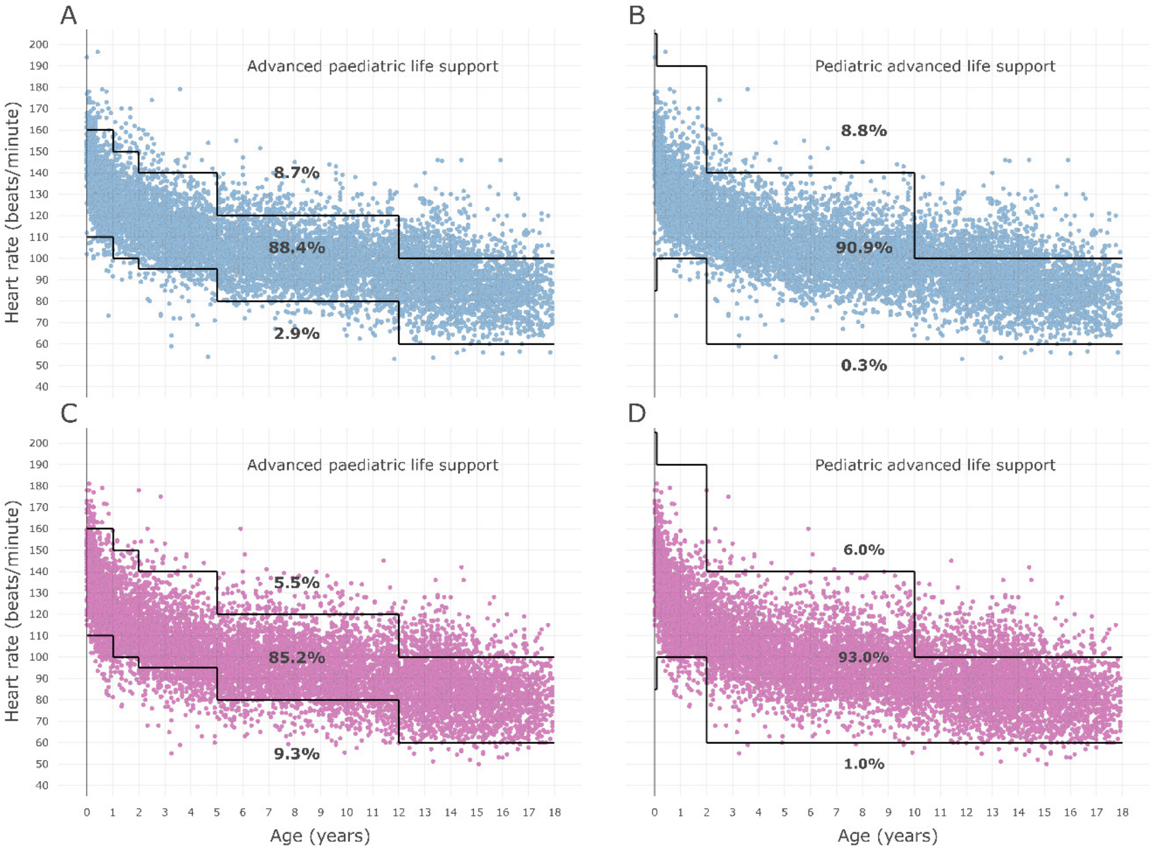

Figure S7. Centile curves for heart rate by age

Centile curves of heart rate by age with visual comparison against the centile curves from advanced pediatric life support (A and C) and pediatric advanced life support (B and D) guidelines. Scatter plots of daytime (A and B) and nighttime (C and D)

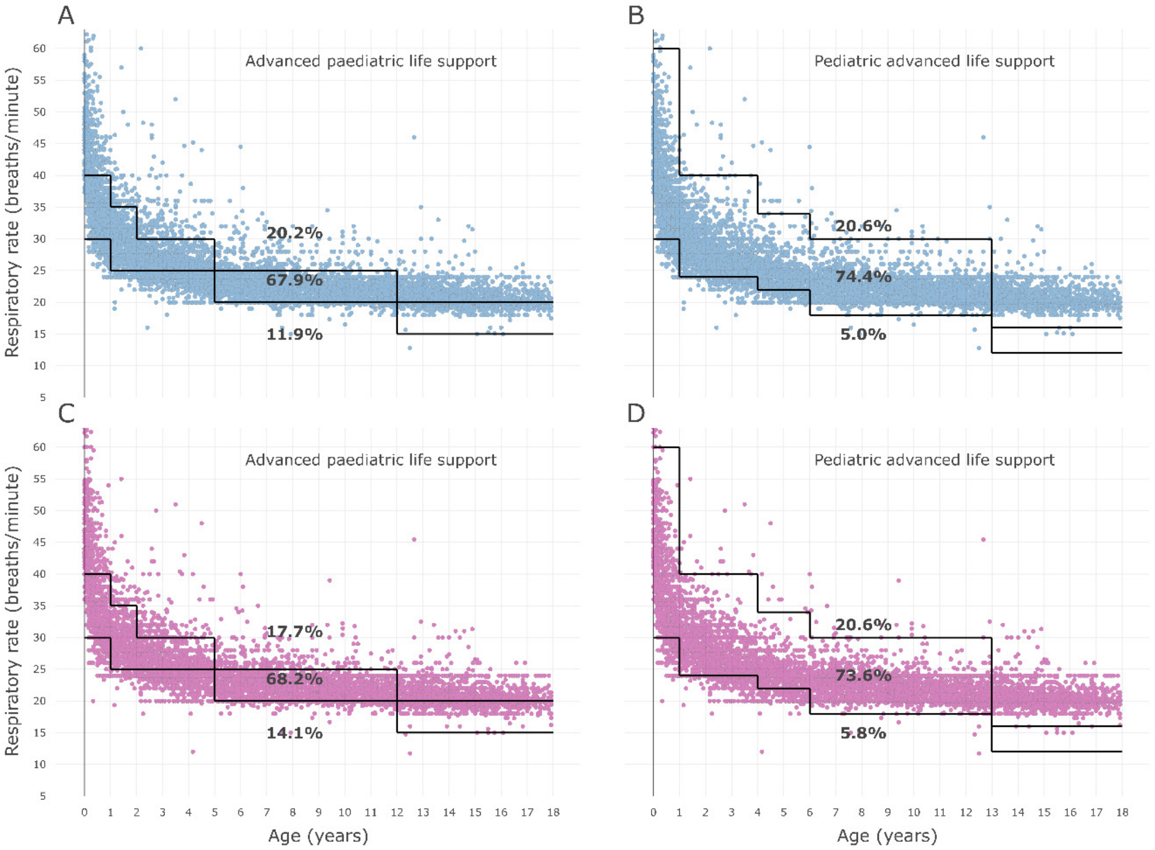

Figure S8. Centile curves for respiratory rate by age

Centile curves of respiratory rate by age with visual comparison against the centile curves from advanced pediatric life support (A) and pediatric advanced life support (B) guidelines. Scatter plots of daytime (A and B) and nighttime (C and D)
